# Looking through goal theories in language learning: A review on goal setting and achievement goal theory

**DOI:** 10.3389/fpsyg.2022.1035223

**Published:** 2023-01-05

**Authors:** Xiaofang Cheng

**Affiliations:** School of Foreign Languages, Technology and Media University of Henan Kaifeng, Kaifeng, Henan, China

**Keywords:** academic motivation, cognitive interface, motivational behavior, achievement goal theory, goal setting, language learning

## Abstract

A growing interest can be seen in the studies on the motivation related to second/foreign learning in recent decades. All in all, research verdicts designate that academic motivation plays a key function in the extent to which students are successful in their research. One of the dimensions of academic motivation is goal orientation, which accounts for why learners carry out achievement activities. This type of goal is indicative of the importance one attaches to success concerning a performance standard. Furthermore, goal setting is deemed as a significant cognitive interface that connects motivation to motivational behavior. Indeed, goal setting is an inseparable part of L2 learning that has caught the attention of many researchers. It functions as a booster of motivation and success in various fields. Goals render the activities purposeful, providing individuals with directions. Moreover, goal drives them to invest more resources and effort, pushing them to persevere in learning. The new versions of motivational theories emphasize social-cognitive components underlying motivated behavior. Therefore, they are more inclusive than traditional ones. Achievement goal theory (AGT) has been developed as a motivation-related theory in recent decades. This theory serves as an effective framework to account for motivation associated with social achievement and learning environments. It also deals with the outcomes concerning cognitive and behavioral aspects. Another theory related to motivation is the Goal setting theory, which functions as a cognitive mediator between motivation and second/foreign learning behavior. It also impacts the students’ application of strategies. Drawing on the recent conceptual developments, this review seeks to make a contribution to the related literature on theories of achievement goals, i.e., AGT and goal setting associated with the L2 context. Such a review has pedagogical implications for EFL stakeholders.

## 1. Introduction

Despite L2 learners’ efforts to make relevant adjustments to the target language, they struggle with multiple challenges related to the complex nature of language and its multilayered structure. In their study, Adwani and Shrivastava (2019) sought to pinpoint the factors contributing to the complexity of SLA. They took into account five variables, one of which was motivation. These authors believe that L2 teaching has turned a blind eye to motivation. They asserted that the entirety of the learning process is impacted by the learners’ motivation. As long as the learners lack motivation, they would experience no vitality (life) and enthusiasm in class (Adwani and Shrivastava, 2019). The study tried to uncover the extent to which motivation as an important variable influences the learning process. Undeniably, motivation involves the individuals’ attempts combined with his/her thirst for acquiring or learning a skill in general and an L2, in particular, driven by the positive perceptions of L2 learning (Achmad and Yusuf, 2016; Usman et al., 2016; Pishghadam et al., 2021). In other words, L2 learning-related motivation is an indicator of the extent to which an individual devotes his/her resources to learning the target language due to the internal driving forces and the enjoyment one gains from doing the task (Rubrecht and Ishikawa, 2012). In Wolters and Rosenthal's (2000) view, motivation serves as a driving force that inspires an individual to engage in an activity or to persevere to achieve a goal. Motivation in the educational context is concerned with the reasons and the justifications a learner has for accomplishing a desirable outcome.

Several factors other than efforts are involved in motivation. The motivated individual allocates time and energy toward the goal, but the individual making effort is not necessarily motivated. Learners’ motivation can impact the learning outcomes in L2 classes (Ulfa and Bania, 2019). It would be insightful to examine how learners make progress in learning EFL/ESL, as well as the failures they have in learning an L2. This is likely to influence their motivation and their learning styles in the context of L2 learning (Dörnyei, 2005). Closely related to motivation constructs, the concept of goal construct and its relations to motivation and L2 learning have caught the attention of researchers in recent years. They assert that goals can serve as the initiator and monitor of the self-regulatory processes associated with L2 learning (Rose et al., 2018; Zheng et al., 2018). Goals are considered a core variable of research conducted on L2 motivation and at the outset, goal theories were introduced in the field of psychology. The goal is concerned with the motives or purposes that an individual has in the learning process. Goal constructs play an essential role in the majority of social-cognitive models of motivation (Dörnyei, 2005). Based on Goal theory, goals are viewed as cognitive manifestations of what people are seeking to fulfill, along with their aims for performing the task. Therefore, goals are considered cognitive constructs, which are deemed to be accessed by a person; unlike the constructs accounted for by psychodynamic theory, goals are conscious motives. Contrary to some models of motivation, goals are not considered dire needs or motives, either (Deci and Ryan, 2000).

According to Locke and Latham (2002), a review of the literature reveals two main goal theories dealt with in motivational studies, namely, goal setting theory and goal orientation theory. The former was put forth by Locke and Latham (1990) in the context of industrial and organizational psychology with a focus on the workplace which is a significant basis of task motivation. Based on this theory, individuals should set goals as human acts are driven by the goal and these goals inspire the actions and behaviors. Following this theory, there are two aspects to goals, namely, internal and external (Dörnyei, 2005). The internal aspect is related to ideas, and the external aspect is related to the object or situation pursued. The internal ideas act as guidelines for accomplishing the goals. According to the goal-setting theory, goals are characterized by the features, which make them different. These features are as follows: specificity and commitment, and difficulty (Locke, 1996). The goal orientation theory contrasts with the goal setting theory; that is, the former emerged as an outcome of the classroom context, which can account for students’ learning and performance (Dörnyei, 2005). Based on this theory, there is a close relationship between a person’s performance and the stated goals. As mentioned by Dörnyei (2005), L2 learning goals are typically known as orientations. However, according to Gardner and Tremblay (1994), there is no explicit connection between ‘orientations’ and various goal theories commonly used in educational psychology.

Achievement goal theory is a motivation-related theory frequently used to examine learners’ motivation and achievement (Hulleman et al., 2010; Wirthwein et al., 2013). For instance, Sins et al. (2008), for example, conducted a study to verify a conceptual paradigm that accounts for the relations between several learner’s variables, including achievement goal orientation, self-efficacy, cognitive processing, and achievement in a specific task involving deep collaboration. The test included a computer-based modeling task involving collaboration aimed at testing the model in terms of mastery-approach goal orientation, performance-evading goal orientation, self-efficacy, and achievement. As expected, the results revealed a significant positive effect of mastery-approach goal orientation on achievement. It is worth noting that this effect was moderated by the participants’ employment of deep processes. The results also showed no significant relationships between performance-avoidance goal orientation and surface processing. There was no correlation between surface processing and achievement, either. In addition, in a study, Juned et al. (2021) investigated the possible relationship between students’ achievement goal, their perceptions of teaching practices, and English academic achievement. The sample of the study was made up of 50 learners who provided information through the questionnaire concerning their achievement goal and how they conceived of English instructors’ teaching practices. The correlation analysis revealed a significant relationship between the mastery approach and English achievement scores. Also, perceived teaching practices indicated no significant relationship between teaching practices and English achievement. The results showed the important role of learners’ motivation in academic achievement in the quality of learning. Lu et al. (2022) used a process-based model to examine the relationship between learning goal orientation (LGO) among university students with their academic performance. A longitudinal study reveals that students who have high LGO in their first month after entering the university generally have higher academic self-efficacy and seek more feedback. Moreover, initial levels of feedback seeking are positively related to academic performance *via* the linear chang e in academic self-efficacy over time.

In the context of educational and motivation psychology, AGT is one of the essential frameworks which helps researchers to gain a conceptualization of learners’ motivation. It also allows them to inspect the role of motivation on L2 learners’ participation, learning, and performance in classes (Senko, 2016; Elliot and Hulleman, 2017). Indeed, this theory integrates several variables including personal and contextual motivational factors (e.g., Kaplan et al., 2002; Urdan and Schönfelder, 2006). Based on this theory, in scholastic circumstances, one’s motivation is influenced by his/her goal orientations, which refer to how achievement is observed and competency is assessed (Dweck and Leggett, 1988). Literature defines achievement goals as the driving forces pushing learners to specifically participate in, focus on, and respond to a wide range of achievement tasks and situations (Meece et al., 2006). The goal plays an essential role in enabling the researchers to work out the learners’ academic achievement and learning (Pintrich and Schunk, 2002).

A mastery goal, also known as a learning goal refers to the learners’ attempts to obtain knowledge or skills. This type of goal has to do with the enhancement of competence through gaining mastery over a task. In contrast, a performance goal, also known as an ego goal, is deemed as a competitive goal by which an individual tries to appear better than others (Pintrich, 2003; Ross, 2008). Indeed, this type of goal involves a comparison between competence and other individuals (Jiang and Zhang, 2021). One can subdivide performance goals into the following: performance-approach and performance-avoidance goals. These two subcategories are concerned with the learners’ beliefs in their ability to do something well or in their beliefs in their inability to do so (Berger, 2009; Van Yperen et al., 2014).

A review of the literature shows that modern motivational theories are different from traditional ones, attaching enormous importance to the social-cognitive components driving motivated behavior (e.g., goal setting and AGT). Notwithstanding many investigations conducted on these theories, as well as their use as a useful motivational technique in various domains, (e.g., Locke and Latham, 1990; Schunk, 1996; Bandura, 1997; Page-Voth and Graham, 1999; Schunk and Ertmer, 1999; Zimmerman, 2008; Mikami, 2017), few studies have sought to examine the effects of these theories on L2 learning and teaching (e.g., Haynes, 2011). The literature provides evidence that these theories are important though they have been disregarded when it comes to FL/SL learning contexts.

## 2. Review of the literature

### 2.1. Goal setting theory

A motivation-related theory called GST (goal-setting theory) was set. From this theory’s perceptive, a goal is viewed as the driving force to power people’s behaviors, which shows the direction an individual needs to take. Based on this theory, behavior or action is driven by an aim, seeking to obtain a particular standard of proficiency, within a pre-determined timeframe (Locke and Latham, 2002). As a result, in its narrow sense, a goal is described as the intended end-state that has a precise and proximal nature regardless of the hidden intents or motives for particular actions. Within 12 years (1990–2002), a multitude of investigations sought to demonstrate that difficulty and the level of goals are the two main factors that determine the level of achievement goals. Indeed, goal difficulty is linearly related to task achievement (Locke and Latham, 2006). Goal setting theory has to do with the relationship between goal determination (goal setting) and behavior, with learners’ selection of goals, the degree of motivation for fulfilling the goals, and the likelihood of the fulfillment of the goals being in the spotlight (Locke and Latham, 2006). This theory is composed of two main components as follows: the individuality and difficulty of the goal, and the effort one needs to fulfill the objectives (Locke and Latham, 2006). As pointed out by Locke and Latham (2002), goal-setting theory refers to a direct relationship between written goals and performance. Indeed, goals give researchers important standards through which they can make a comparison between work. Thanks to these efforts, the individual receives feedback on his/her competence, which contributes to the enduring motivation for learning. Based on GST, the individual makes the most effort in the face of moderately difficult tasks. Such goals, i.e., challenging ones, inspire individuals to do their best (Locke and Latham, 2002). Goal setting increases the learners’ motivation, helping them to reinforce their self-regulation (Locke and Latham, 2006). This, in turn, empowers them to be committed to learning. Scholars have concluded that learners can use goal setting as an effective inspiring tool, which contributes to positive attributes, including intrinsic motivation, positive self-image, and academic performance (e.g., Latham and Locke, 2006; Schunk, 2009).

### 2.2. Achievement goal theory

Achievement goal theory, which is also known as normative goal theory, has been cited as one of the outstanding theories of motivation developed in a social-cognitive setting. This theory places emphasis on learners’ intentions for persevering in various learning activities (Meece et al., 2006). Drawing on the conceptualizations of motivation and achievement-oriented behaviors, AGT theory focuses on the driving forces pushing a student to achieve an already stated outcome. It also has to do with the goals that drive the learners to display achievement-oriented behaviors (Maehr and Zusho, 2009). Furthermore, as mentioned by Pintrich (2000), present theories about goal orientation deal with the standards or criteria constructed by the learners thereby they can assess the extent to which they are successful in performing a task. One should make a distinction between this theory and the other social-cognitive theories associated with the construct of motivation (e.g., Expectancy-value and self-efficacy). Indeed, AGT theory attaches great importance to the goals set by individuals during the development of their competence (demonstration or development of competence). In this context, the perceptions of one’s capability or the attributions made by the learners regarding their academic performance are very important (Meece et al., 2006).

Achievement goal theory is closely concerned with two goals; mastery and performance (Kaplan and Maehr, 2007). The mastery goals are adaptive and efforts should be made to enhance such goals at both the learner and classroom levels. The mastery goals are concerned with enhancing competence through hard work, while the performance goal is related to demonstrating competence in a domain, which allows the individual to outperform others. Considering the emphasis placed on the attainment of competence, both goals fall into one category, i.e., competence-related goals (Elliot and Church, 1997). Mastery goals belong to the value-intrinsic quadrant, while performance goals belong to the value-extrinsic quadrant. Mastery goals emanate from the internal driving forces though they can be impacted by instructors’ behaviors. In their pursuit of mastery goals, learners seek to increase and improve their competence. Meanwhile, they do their best to understand and master the learning material, obtain knowledge, or develop a new skill (Covington, 2000). External variables (e.g., instructor’s perceptions or family’s behaviors) can impact performance goals. In their pursuit of performance goals, learners are obsessed with how others judge or compare them in terms of their ability. They want to know whether others perceive them favorably, seek to do better than others, and demonstrate one’s capabilities to others. They are always seeking others’ positive comments on their performance (Covington, 2000). Having a performance goal in their minds, people compare their competence against an interpersonal standard. Put it another way, they compare themselves to others.

A positive relationship exists between mastery goals and several learner variables including, self-image, task value, the type of cognitive strategy used by the individual, and self-controlled learning (Ross, 2008). On the contrary, other types of goals, namely, performance-avoidance ones are destructive, dealing with the prediction of adverse learning outcomes (Hulleman et al., 2010). In their study, Deci and Ryan (2000) showed an association between mastery goals and intrinsic motivation, whereas they found a relationship between performance approach and extrinsic motivation. As a result, the learners having a high level of intrinsic motivation are expected to set mastery goals, while the learners with a high degree of extrinsic motivation set performance goals.

## 3. Conclusion, implications, and future direction

The impact of the learning environment and learner’s traits on performance and learning outcomes can be explained in the light of goal orientation (Pintrich and Schunk, 2002). The features of the goals set by the learners can potentially impact their cognition, emotions, and behavior in various contexts (e.g., performance-based assessment and testing). Although learners’ successful performance is largely assessed in terms of scores and grades, teachers can channel their teaching efforts in a positive direction by being aware of their student’s goal orientation. This approach would result in a positive mood and atmosphere in the L2 classroom. Setting specific goals is impacted by a variety of factors (e.g., sense of competence/ability and social variables). The goals also can be seen as a window into the effect of various classroom structures and school settings on learners’ level of motivation and learning. Instead of emphasizing perceived capability and causal attributions, the goal theories associated with motivation deal with the different types of goals people seek to fulfill in achievement situations. Indeed, behavior is regarded by goal theorists as structured, focused, and directed toward the fulfillment of particular goals (Pintrich and Schunk, 2002). The AGT is deemed an essential approach to examining the impact of goal structures and class setting on the learners’ level of motivation and achievement (Anderman and Wolters, 2006). This theory deals specifically with goals individuals set for the development of competence. It also concerns the learners’ motives and intentions for taking part in various learning tasks. Based on this theory, goals signal the stated purposes, motives, and/or the purposes which drive people to get involved in achievement activities. Various goals influence learners’ cognitive, affective, and behavioral aspects differently. The scholars working in the area of achievement goals concentrate specifically on the aim associated with the enhancement or demonstration of competence. Indeed, goal setting can be viewed as a useful tool that one can use in the EFL classroom to motivate learners to improve their performance. This theory can be especially used in environments where there is a strong focus on students’ performance on tests and motivation plays an essential role. Goal setting allows the learners to control their own learning by setting targets perceived by them to be relevant to L2 success.

It follows that goal setting can be used as an operational method teachers may use in the EFL classroom for the purposes of motivating learners to improve their L2 performance. Goal setting is expected to be useful in contexts where great importance is attached to learner performance on tests so that their successful test performance is highly motivating to them. Indeed, the teacher must make it clear to the students that goal setting is mainly aimed at driving students to go beyond their current limitations; that is, they do not have to compete with their peers, particularly, if test scores function as criteria for the assessment of goal attainment. Moreover, following the goal-setting theory, individuals who show a strong commitment to challenging yet, achievable goals have a successful performance on goal-relevant tasks. Indeed, people whose goals are challenging and achievable make more arduous efforts compared to individuals with no such goals. Based on this theory, being committed to a challenging yet achievable goal is essential for improving motivation and performance. Goal commitment is inevitable in that a motivated individual makes more effort to fulfill the goal, which, in sequence, results in better performance. Research findings reveal that a strong commitment to a goal results in higher performance on the part of people and teams (Brown and Latham, 2000; Knight et al., 2001). Setting a learning aim can be very beneficial to the students, and they are deemed an effective way to enhance performance (Burton et al., 2010). Based on the review of the literature, it can be concluded that in line with Goal’s theories, all actions are driven by a purpose. Setting a goal based on a choice is a requirement for the occurrence of action and, goals must be sought with effort and seriousness. Therefore, setting the appropriate goal as well as the provision of timely and specific feedback can bring higher achievement, successful performance, a high degree of self-efficacy, and self-regulation (Schunk, 2009). This paper seeks to provide insights into how goals and goal-oriented efforts play a role in successful L2 learning. Due to the efforts made by students, the goal-related issues have important implications for the conceptual development undertaken by goal theorists; moreover, they can be helpful to educational practitioners who seek to improve the quality of education.

Given that goal setting has been shown to function as a powerful strategy to enhance L2 acquisition, L2 educators and instructors should develop an intervention program to facilitate learners’ goal setting in L2 classrooms. Such programs encourage the learners to set challenging goals (demonstrating goal difficulty), divide them into practical action plans (a reflection of goal specificity), and reach the collective outcomes in an L2 setting. As revealed by related literature (VandeWalle and Cummings, 1997), goal orientation is composed of both dispositional and situational elements. This implies that knowledge of how goal orientation functions make it possible for the teachers to develop strategies to determine learners’ goal orientations effectively. This review of related literature shows that an examination of the impact of goals and relevant processes associated with L2 learning can yield positive outcomes, including finding about L2 learners’ motivation. This helps them to have successful experiences regarding L2 learning. In line with the relationship between goal theories and L2 education, the following recommendations can be taken into account by L2 researchers and practitioners who find goal constructs in L2 education settings appealing and instructive. It is recommended that L2 learning researchers take into account the important role played by these theories in L2 acquisition. Accordingly, it is suggested that prospective studies examine the impact of each one of the theories on L2 learning and progress. Based on AGT, achievement goals have a forward-looking perspective, and are deemed as cognitive manifestations of intended outcomes (Hulleman et al., 2010). Indeed, such goals channel individual behavior in a targeted way. Here, the individual’s perceptions of competence play an essential role. As a result, achievement goals provide scholars and practitioners with a way to work out the reasons for the learners’ engagement in achievement settings.

It is revealed that there are several benefits associated with adopting the mastery goal approach: learners can become self-confident and gain satisfaction with learning tasks. This approach places emphasis on enhancing learners’ abilities, acquisition of new skills, fulfillment of challenging tasks, and benefitting from learning resources. A performance goal perceptive focuses on the significance of possessing a higher level of abilities compared to others. Learners’ achievement is dependent on whether they outperform others, or whether they surpass the normative performance standards. Learners who focus on goal orientation seek to acquire the prerequisites including new knowledge to gain mastery over the skills, along with boosting their ability. These learners tend to do challenging tasks, making use of learning strategies for achieving positive outcomes. They always persevere and make efforts, having positive perceptions of learning and spending a rather long time to accomplish the tasks enthusiastically (Church et al., 2001). To them, intelligence is not a fixed ability. This implies that effort and hardworking, as well as self-regulation, are the main features of mastery-oriented learners (Anderman and Patrick, 2012).

This study has several implications for the stakeholders in the setting of L2 teaching and learning. L2 teachers need to take into consideration the individual differences in their classes. These differences are closely related to the degree of motivation, particularly, regarding the L2 learning process. Put it another way, the current review reaffirms the essential role played by motivation in realizing L2 learning success. Other players such as material developers and educators are advised to think through motivation an essential contributor to L2 learning. Indeed, motivation can play a role in preparing the learners. Therefore, teachers do well to use the relevant instruction strategies and textbooks to improve learners’ motivation. Moreover, goal setting and goals can be influential in the context of L2 learning and teaching. They can induce positive attitudes among the learners. That is, this study reached the conclusion that L2 learners with clearly set goals for learning are more likely to obtain their academic goals than students with no goals. Consequently, educators need to raise the learners’ awareness about the essential contribution of goals to L2 learning. This allows them to keep up with their goals during their learning. In addition, goals function as guidelines showing students the right learning path. Learners need to use strategies should they fulfill their goals. Despite the positive outcomes concerning challenging goals (such as actualizing the learners’ potential), it is helpful to set goals given the students’ present level of ability. This is because failure to take into account the abilities may result in disappointment and frustration, which, in turn, leads to a decrease in motivation. As a result, when it comes to L2 motivation, goal setting is very essential and it cannot be disregarded. Both teachers and learners can set goals. As far as teenager L2 learners are concerned, they can set their goals by following the guidelines provided by their teachers or parents. Goals should be specific because students need to grasp the significance of goals, as well as the importance of making efforts to achieve the goals. Given the positive effect of students’ engagement in goal setting on their performance, teachers should emphasize implicating them in the development of goal setting (Azevedo et al., 2002). This is because learners find the goal set by themselves more relevant and meaningful.

Thanks to the development of AGT, researchers have access to an effective explanatory framework according to which goals are deemed as changeable personal traits; moreover, as pointed out by Schunk et al. (2008), goals are shaped under the influence of both teacher and learning context. ESL instructors always seek to devise ways to enhance and assess L2 instruction and learning in schools. The aim is to make and maintain learners motivated in English learning. The prospective research can shed light on the types of teaching strategies teachers use to improve learners’ achievement goals and academic success. Prospective studies can focus on the empirical examinations of the following: the impact of multiple types of learning goals and how they can be integrated with performance goals (e.g., learning goals should be prioritized followed by performance goals), various kinds of goal framing, the potential relation between goals and cognition (such as goal classification, and macro goal research).

## Author contributions

The author confirms being the sole contributor of this work and has approved it for publication.

## Conflict of interest

The author declares that the research was conducted in the absence of any commercial or financial relationships that could be construed as a potential conflict of interest.

## Publisher’s note

All claims expressed in this article are solely those of the authors and do not necessarily represent those of their affiliated organizations, or those of the publisher, the editors and the reviewers. Any product that may be evaluated in this article, or claim that may be made by its manufacturer, is not guaranteed or endorsed by the publisher.
